# Early-Life School, Neighborhood, and Family Influences on Adult Health: A Multilevel Cross-Classified Analysis of the Aberdeen Children of the 1950s Study

**DOI:** 10.1093/aje/kwu110

**Published:** 2014-06-12

**Authors:** Ruth Dundas, Alastair H. Leyland, Sally Macintyre

**Keywords:** family, median odds ratio, neighborhood, schools

## Abstract

Lifetime exposures to adverse social environments influence adult health, as do exposures in early life. It is usual to examine the influences of school on teenage health and of adult area of residence on adult health. We examined the combined long-term association of the school attended, as well as the area of residence in childhood, with adult health. A total of 6,285 children from Aberdeen, Scotland, who were aged 5–12 years in 1962, were followed up at a mean age of 47 years in 2001. Cross-classified multilevel logistic regression was used to estimate the associations of family, school, and area of residence with self-reported adult health and mental health, adjusting for childhood family-, school-, and neighborhood-level factors, as well as current adult occupational position. Low early-life social position (as determined by the father's occupational level) was associated with poor adult self-rated health but not poor mental health. There were small contextual associations between childhood school environment (median odds ratio = 1.08) and neighborhood environment (median odds ratio = 1.05) and adult self-rated health. The share of the total variance in health at the family level was 10.1% compared with 89.6% at the individual level. Both socioeconomic context and composition in early life appear to have an influence on adult health, even after adjustment for current occupational position.

***Editor's note:*** *An invited commentary on this article appears on page 208*, *and the authors’ response appears on page 213.*

Lifetime exposures to adverse social events have an influence on adult health and disease. The evidence also points to exposures in early life having a lasting impact on adult health outcomes.

It is usual to examine contemporaneous school effects on educational outcomes (1, 2) and current health (3, 4). Most studies of school effects are concerned with educational outcomes. Fewer studies address the role of school on health outcomes. It is unclear whether there are any long-lasting effects of the school attended. A previous study examining school and individual social measures found that school had a small effect on longer-term health behaviors and outcomes (5). Children spend a large part of their time in school; it is analogous to the workplace for adults. Both the cultural environment of the school and the curriculum may have an impact on the health and health behaviors of children (3, 4), thus influencing adult health.

Most research on neighborhood and health focuses on current area of residence and current health status. Significant associations between the social circumstances of areas and self-rated health have been observed; studies have shown that areas with poor social conditions are associated with poor self-rated health (6), and areas with greater deprivation are associated with higher risk of death (7). Studies that have examined area of residence over the life course and its effect on the risk of death have found mixed results (8); 1 study found that poor area conditions in childhood were associated with higher risk of death 50 years later (9), whereas another study reported that area of residence over the life course had little influence on subsequent risks of death and morbidity (10).

Studies that have attempted to disentangle the contemporaneous influences of neighborhood and school have found significant variation at the school level, which was greater than that at the area level, on health behaviors at ages 13–15 years (11). Rasbash et al. (2) found that 8.5% of variation in educational performance was at the primary school level, and a smaller amount was at the area level. Most of the variance was at the individual and family levels.

There is a need to extend previous work (5) by examining data on school and residential area in childhood to make a more complete analysis of the association of childhood social position on adult health. Multilevel models enable separation of the contribution that each environment makes to the outcome of interest (12). It is important to interpret both measures of variance and measures of association. Doing so can provide a better understanding of the patterning of health and health inequalities (13). If the contexts under investigation (e.g., area, school, and family) are relevant for individual health, then a meaningful share of the total individual variation can be expected at the context level. Moreover, if this share is meaningful, further factors that contribute to the variation between specific contexts may be identified, enabling specific contextual interventions to be planned at these levels. On the other hand, if the share of variation is low, then it is better to target the whole population rather than the specific contexts.

Here, we use data from a longitudinal study of children born in 1950–1956 in Aberdeen, Scotland, who were followed up in 2001 at ages 44–51 years. We use multilevel models to partition the variance of the environment in which the children lived to identify which part—individual, family, school, or childhood area of residence—has the greatest association with adult health, thus exploring possible explanations for the association of early-life social circumstances and adult health.

## METHODS

**Figure 1.**
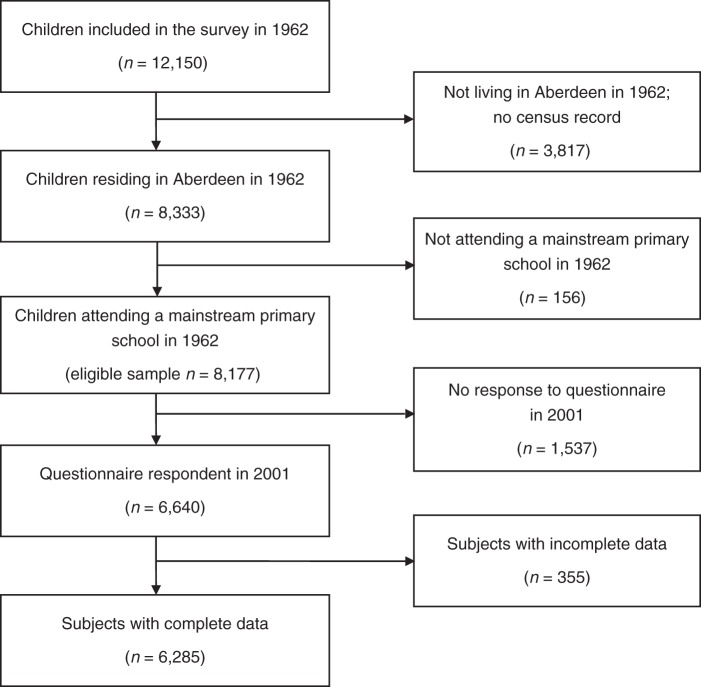
Flow chart of participants in the Aberdeen Children of the 1950s Study who were included in the analysis. The eligible sample is those who participated in the 1962 survey and were attending a mainstream primary school and residing in Aberdeen, Scotland. The proportion of eligible subjects with complete data is 76.9%.

The Aberdeen Children of the 1950s Study has been described elsewhere (14, 15). It is a survey of the total population of all children aged 5–12 years, born in Aberdeen, Scotland, in 1950–1956 who attended primary school in Aberdeen in December 1962. The aim of the original survey was to ascertain the population prevalence of learning disability. The cohort was revived in 1998, and the original 12,150 subjects were traced through the United Kingdom's National Health Service Central Register. Vital status and whereabouts were established for 98.5% of the cohort with follow-up questionnaires mailed to 11,321 surviving participants in 2001 (16). Figure 1 shows inclusion and exclusion criteria for the present study.

### Childhood measures

The data used to describe individual and family socioeconomic circumstances in childhood were collected in the 1962 survey. They are family size (≤3 or >3 children in family), father's occupational class (each job categorized as professional, managerial, skilled nonmanual, skilled manual, partly skilled, or unskilled), home ownership, and overcrowding (i.e., >1 person per room) in the home. These variables are referred to as individual-level measures; children from the same families are in the same sibling group and have the same values for each measure. To allow for this in the analyses, sibling group was included as a level in the models.

### Specific contextual effects of childhood school- and area-level data

Specific contextual effects display the association between variables measured at the higher levels and the individual-level outcome (17). School socioeconomic status (SES) was measured by an aggregated variable comprising the proportion of children in each school with fathers in professional and managerial occupations. The aggregated school variable was calculated using the original 12,150 subjects from the 1962 survey.

Area of residence in 1962 was linked to information from the 1961 census. This information is available at the enumeration district level. Enumeration districts were the geographical areas that enumerators covered in the 1961 census. There were 10,400 enumeration districts in Scotland, each with an average population of 500 individuals living in 150 households (18, 19). This was used as a proxy for neighborhood; the neighborhood variables used were the proportion of homeowners in the enumeration district and the proportion of homes in the enumeration district that had the following 4 amenities: hot water, cold water, fixed bath, and toilet. These were categorized into quartiles for the analyses.

### Adult social position

Adult health is affected by adult social position. Childhood SES is strongly associated with adult SES. To account for this, we used 2 measures to describe adult individual socioeconomic circumstances on the basis of the 2001 survey: adult occupational class and educational level (no formal qualifications, basic formal qualifications, or higher-level formal qualifications) as measured by age upon leaving secondary school (<16 years, 16 years, or >16 years).

### Outcome measures

We examined the following 2 self-reported health outcomes from the follow-up questionnaire: self-rated health and self-rated mental health in adulthood. For self-rated health, respondents were asked, “Over the last 12 months, would you say that your health on the whole has been excellent, good, fair, or poor?” The responses “excellent” and “good” were grouped together, as were “fair” and “poor.” Self-rated health is highly associated with subsequent risk of death (20), as well as with current comorbidity and clinical disease (21). The outcome used here is fair or poor health (referred to as “poor health”). Respondents' mental health was measured by using a 4-item version of the General Heath Questionnaire (GHQ-4) (22, 23). The 4 questions were as follows: In the past few weeks have you been 1) … feeling reasonably happy, all things considered; 2) …able to enjoy your normal day-to-day activities; 3) …losing confidence in yourself; and 4) …feeling unhappy and depressed (Cronbach α = 0.83). GHQ-4 caseness was recorded as “yes” if any of the responses was adverse and “no” otherwise (23).

### Statistical methods

**Figure 2.**
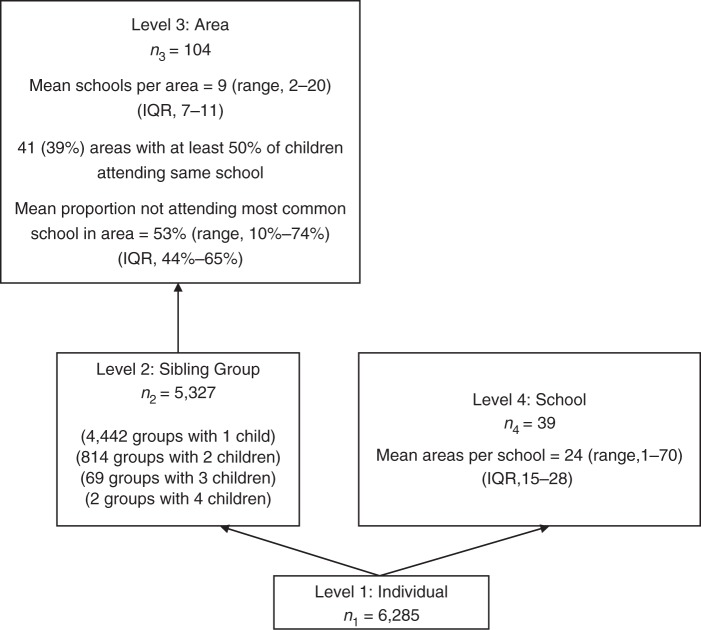
Diagram for the 4-level classification models of individuals, sibling groups, areas, and schools in the Aberdeen Children of the 1950s Study. Areas are enumeration districts from the 1961 census, and schools are primary schools in Aberdeen, Scotland, in 1962. Individuals are nested within sibling groups, and sibling groups are nested within areas. One cross-classification arises from individuals from the same sibling group not attending the same school. This cross-classification does not exist for sibling groups containing 1 child nor for sibling groups containing 4 children, because all 4 children in these sibling groups attended the same school. The cross-classification occurs for sibling groups of 2 or 3 children; 130 (16%) sibling groups of 2 children attended different schools, and 13 (19%) sibling groups of 3 children attended different schools. There is a further cross-classification of areas and schools where individuals from the same area attend different schools. None of the 104 areas was nested within a school; all areas had some children who attended different schools. The absence of arrows displays these cross-classifications; where an arrow exists, it displays the typical nested multilevel model structure (25). IQR, interquartile range.

Individuals are nested within neighborhoods and within schools. Each of these contexts may condition individual-level variation due to unmeasured factors. This dependence of the individual outcome within contexts motivates the application of multilevel regression analyses, and it is also a source of substantive information; the higher the dependence the greater the relevance of the context for understanding individual outcomes. A single school may contain children from many neighborhoods, and children from 1 neighborhood may attend different schools. At the time of this study in the 1960s, children were not necessarily assigned to schools on the basis of their residential addresses. The schools are not nested within neighborhoods, and neighborhoods are not nested within schools. For this reason, cross-classified multilevel logistic regression models were used (24). Cross-classified models allow for the estimation of the variance at each level, taking into account the complex hierarchical structure. Sibling group was also included as a level because there were children from the same families in the study. There was further cross-classification between sibling group and schools because siblings do not necessarily attend the same school (Figure 2).

The 4-level cross-classified logistic regression models were fitted in WinBUGS (25) using the Markov chain Monte Carlo method, running 2 chains of 50,000 iterations following a burn-in of 20,000. For the fixed effects (measures of association), a normal prior was used, and a half-normal prior was used for the square root of the variance.

For both outcomes—self-rated health and mental health—the analytical strategy was to calculate the changes in variance and median odds ratios resulting from the addition of the different specific and general contextual effects (i.e., individual, neighborhood, and school contexts) and the change caused by the addition of adult mediating factors. We first fit a model containing age and sex (model 1). We then fit a model to examine individual childhood factors (model 2), then the neighborhood and school SES in combination with individual factors (model 3). Finally, we fit a model to adjust for mediating variables (model 4). Most individuals were in single-sibling groups, so the variance at the sibling level for these children is, by definition, included with the individual variance. Estimating a “variance” for them at this level may not be appropriate. To account for this in the analyses, we estimated only the sibling variance for those in sibling groups of more than 1. The variance at the sibling level is reparameterized to reflect the fact that most sibling groups contain only 1 individual (2). Appendix 1 details the formulae for these models.

Because the focus of the analysis was on variances, we assessed the relative importance of the general contextual effects of schools, neighborhoods, and sibling groups using the following measures (17): the median odds ratio (26, 27) and the variance partition coefficient (VPC) (28, 29). The median odds ratio is on the same scale as an odds ratio and is interpreted as the median value of the odds ratios between individuals from units at high or low risk when randomly choosing 2 individuals from different units. In these analyses, this is the odds of having poor self-rated health or low GHQ-4 score that are determined by unexplained factors at the sibling, area, and primary school levels. The formulae and interpretation of the VPCs are in Appendix 2. The VPC is the share of the total individual variance attributable to a given level.

## RESULTS

Questionnaires were received from 3,039 men and 3,246 women (77% response rate). They had a mean age of 47 years. There were 39 schools with an average of 163 respondents each (range, 4–400) and 104 enumeration districts with an average of 61 respondents per district (range, 11–211).

Table 1 shows descriptive information for the individual child and adult variables and prevalences and interquartile ranges for the contextual school and neighborhood factors. A fifth of respondents had both poor self-rated health and GHQ-4 scores indicating poor health. The median proportion of households lacking all 4 amenities (hot water, cold water, bath, and toilet) in a neighborhood was 30% (interquartile range, 8%–54%), and the median proportion of households with occupants who did not own their own home was 86% (interquartile range, 72%–96%).

**Table 1.** Summary Characteristics for the 6,285 Subjects in the Aberdeen Children of the 1950s Study With Follow-up in 2001

Characteristic	No.	%	Mean (SD)	Median (IQR)
Poor self-rated health	1,251	19.7		
GHQ-4 score indicating poor health^a^	1,264	20.0		
Individual level				
Sex				
Men	3,067	48		
Women	3,268	52		
Overcrowding^b^	3,760	59.4		
No home ownership	5,233	82.6		
Family size >3 people	1,981	31		
Occupational class				
I (Professional) or II (managerial)	649	10.2		
III-NM (Skilled nonmanual)	2,404	37.9		
III-M (Skilled manual)	1,223	19.3		
IV (Partly skilled)	862	13.6		
V (Unskilled)	924	14.6		
Other	273	4.3		
Neighborhood level				
Proportion lacking 4 amenities^c^				30 (8–54)
Proportion without home ownership^d^				86 (72–96)
School level				
Proportion of fathers in occupational class I or II^e^				8 (3–14)
Adult factors				
Age			47 (1.5)	
Occupational class				
I or II (Professional or managerial)	2,627	41.5		
III-NM (Skilled nonmanual)	1,586	25.0		
III-M (Skilled manual)	1,147	18.1		
IV or V (Partly skilled or unskilled)	917	14.5		
Other	58	0.9		
Age on leaving school, years				
<16	2,938	46.4		
16	1,461	23.0		
>16	1,936	30.6		

Abbreviations: GHQ-4, 4-item version of the General Health Questionnaire; IQR, interquartile range; SD, standard deviation.

^a^ Mental health was assessed using the GHQ-4, which asks whether, in the past few weeks, respondents have been 1) feeling reasonably happy, all things considered; 2) able to enjoy your normal day-to-day activities; 3) losing confidence in yourself; and 4) feeling unhappy and depressed.

^b^ More than 1 person per room in the home.

^c^ Proportion of homes in the enumeration district without the following 4 amenities: hot water, cold water, fixed bath, and toilet.

^d^ Proportion of people who did not own their own homes in the enumeration district.

^e^ Proportion of children with fathers from professional and managerial occupational classes.

Table 2 presents the results of 4 models analyzing the odds of poor self-rated health. Model 2 shows that family size and father's occupational class were associated with poor adult self-reported health; people from large families and those from families of lower occupational class were more likely to report poor health. The individual measures of overcrowding and living in a home not owned by the family were not associated with poor adult health. Model 3 included measures at the school and neighborhood levels. Adults who attended a school comprising a higher proportion of children with fathers from professional and managerial occupational classes were less likely to have poor self-rated health, and those who lived in neighborhoods with more households lacking the 4 amenities were more likely to have poor self-rated health. The linear dose-response relationship with lack of the 4 amenities remained when this variable was included in the model as a continuous measure (results not shown). After further adjustment for adult SES (in model 4), the individual measures of family size and paternal occupational class, as well as neighborhoods with more households lacking the 4 amenities were still associated with poor adult health. Adult SES was related to poor adult health, with those in a lower occupational class as adults and those with fewer years of education reporting poorer health. Adjustment for adult SES had little effect on the associations of childhood measures of occupational class with adult health.

**Table 2.** Odds Ratios and Median Odds Ratios for Poor Self-Rated Health for the Models Adjusting for Childhood and Adult Variables Among 6,285 Subjects in the Aberdeen Children of the 1950s Study, Aberdeen, Scotland, Followed-up in 2001

Variable	Model 1		Model 2		Model 3		Model 4	
	Odds Ratio	95% CI	Odds Ratio	95% CI	Odds Ratio	95% CI	Odds Ratio	95% CI
*Individual Factors*								
Sex	0.83	0.73, 0.94	0.83	0.73, 0.95	0.83	0.73, 0.95	0.88	0.76, 1.01
Age	1.06	1.02, 1.11	1.06	1.01, 1.10	1.05	1.01, 1.10	1.04	1.00, 1.09
Overcrowding^a^			1.00	0.87, 1.16	0.99	0.86, 1.15	0.99	0.85, 1.16
No home ownership			1.15	0.91, 1.45	1.00	0.78, 1.30	1.01	0.79, 1.32
Family size >3 children			1.38	1.20, 1.60	1.38	1.19, 1.59	1.19	1.03, 1.37
Occupational class								
III-NM (Skilled nonmanual)			1.52	1.14, 2.07	1.42	1.06, 1.93	1.24	0.93, 1.68
III-M (Skilled manual)			1.61	1.17, 2.24	1.47	1.07, 2.06	1.17	0.86, 1.63
IV (Partly skilled)			1.99	1.43, 2.79	1.80	1.29, 2.54	1.36	0.98, 1.91
V (Unskilled)			2.23	1.60, 3.13	2.00	1.44, 2.83	1.47	1.06, 2.05
Other			2.02	1.35, 3.04	1.82	1.21, 2.73	1.49	0.99, 2.25
*Specific Contextual Effects*								
Lacking 4 amenities^b^								
Quartile 2					1.35	1.04, 1.74	1.22	0.95, 1.59
Quartile 3					1.18	0.96, 1.45	1.11	0.90, 1.37
Quartile 4					0.94	0.77, 1.15	0.92	0.75, 1.13
Low home ownership^c^								
Tertile 2					1.27	0.98, 1.63	1.18	0.93, 1.49
Tertile 3					1.27	1.02, 1.58	1.19	0.96, 1.46
School occupational class^d^								
Quartile 2					1.22	0.97, 1.53	1.03	0.82, 1.29
Quartile 3					1.28	1.00, 1.62	1.12	0.88, 1.41
Quartile 4					1.25	0.98, 1.58	1.10	0.87, 1.38
*Adult Factors*								
Adult occupational class								
III-NM (Skilled nonmanual)							1.25	1.03, 1.50
III-M (Skilled manual)							1.52	1.24, 1.87
IV (Partly skilled)							2.40	1.96, 2.93
V (Unskilled)							0.67	0.25, 1.50
Age on leaving school, years								
<16							1.62	1.33, 1.97
16							1.25	1.01, 1.54
*General Contextual Effects*								
Sibling level								
Variance	0.480		0.351		0.350		0.260	
MOR	1.93	1.37, 2.48	1.76	1.15, 2.28	1.75	1.16, 2.31	1.62	1.06, 2.19
VPC (%)	12.4		9.6		10.1		7.7	
Neighborhood level								
Variance	0.005		0.004		0.003		0.003	
MOR	1.07	1.00, 1.20	1.06	1.00, 1.18	1.05	1.00, 1.17	1.05	1.00, 1.17
VPC (%)	0.1		0.1		0.1		0.1	
School level								
Variance	0.073		0.017		0.006		0.004	
MOR	1.29	1.18, 1.46	1.13	1.01, 1.26	1.08	1.01, 1.21	1.06	1.00, 1.18
VPC (%)	1.9		0.4		0.2		0.1	
DIC	6,155.91		6,117.18		6,111.67		5,963.47	

Abbreviations: CI, credible interval; DIC, deviance information criterion; MOR, median odds ratio; VPC, variance partition coefficient.

^a^ More than 1 person per room in the home.

^b^ Proportion of homes in the enumeration district without the following 4 amenities: hot water, cold water, fixed bath, and toilet.

^c^ Low proportion of people who owned their own homes in the enumeration district.

^d^ Proportion of children with fathers from professional and managerial occupational classes.

The median odds ratios calculated from the variances are fairly high for the unadjusted model (model 1) at the sibling level (median odds ratio = 1.93) and school level (median odds ratio = 1.29). The sibling-level median odds ratio was attenuated slightly as more variables were included (models 2–4). The school-level median odds ratio decreases when individual factors are included in the model and decreases further when school and adult factors are included (models 3 and 4). The area variances are much smaller than the school variances in models 1 and 2; however, after adjustment for the individual childhood factors, specific contextual effects, and mediating variables, the unexplained variance at school and area levels is small. VPCs at each level for self-rated health for all models show that the proportion of the unexplained variation at the individual level is considerably larger than at the sibling, school, and area levels. Such findings are common in multilevel logistic regression (27). The VPCs suggest that approximately 8%–12% of the unexplained variation in all models is attributable to differences between siblings.

In contrast to self-rated health, GHQ-4 score shows little relationship with any of the social position measures, either at the individual level or area and school levels (Table 3). The median odds ratios for the variances show the sibling level to be more important than area or school level for predicting poor mental health. The sibling-level median odds ratio is attenuated slightly following adjustment for social position. The school-level median odds ratio does not change, but the variance decreases when individual adult factors are included in the model. The area variances do not change with any of the adjustments.

**Table 3.** Odds Ratios and Median Odds Ratios for Mental Health^a^ for the Models Adjusting for Childhood and Adult Variables Among 6,285 Subjects in the Aberdeen Children of the 1950s Study, Followed-up in 2001

Variable	Model 1		Model 2		Model 3		Model 4	
	Odds Ratio	95% CI	Odds Ratio	95% CI	Odds Ratio	95% CI	Odds Ratio	95% CI
*Individual Factors*								
Sex	0.69	0.61, 0.79	0.69	0.61, 0.79	0.69	0.61, 0.79	0.71	0.61, 0.82
Age	0.97	0.93, 1.02	0.97	0.93, 1.01	0.97	0.93, 1.01	0.97	0.92, 1.01
Overcrowding^b^			0.99	0.86, 1.14	0.98	0.85, 1.14	0.98	0.85, 1.13
No home ownership			1.10	0.88, 1.37	0.98	0.77, 1.26	0.99	0.80, 1.31
Family size >3 children			1.21	1.04, 1.39	1.19	1.03, 1.38	1.11	0.96, 1.29
Occupational class								
III-NM (Skilled nonmanual)			0.94	0.73, 1.22	0.91	0.71, 1.18	0.86	0.67, 1.10
III-M (Skilled manual)			0.86	0.65, 1.14	0.82	0.62, 1.10	0.75	0.56, 0.99
IV (Partly skilled)			1.06	0.79, 1.43	1.01	0.75, 1.37	0.90	0.67, 1.20
V (Unskilled)			1.04	0.77, 1.40	0.99	0.73, 1.34	0.86	0.64, 1.15
Other			1.08	0.74, 1.57	1.03	0.70, 1.50	0.94	0.64, 1.37
*Specific Contextual Effects*								
Lacking 4 amenities^c^								
Quartile 2					1.17	0.91, 1.50	1.13	0.88, 1.43
Quartile 3					1.19	0.96, 1.50	1.17	0.94, 1.45
Quartile 4					1.04	0.80, 1.34	0.99	0.76, 1.28
Low home ownership^d^								
Tertile 2					1.07	0.87, 1.33	1.05	0.84, 1.30
Tertile 3					1.00	0.81, 1.23	0.98	0.80, 1.22
School occupational class^e^								
Quartile 2					1.09	0.85, 1.38	1.01	0.79, 1.28
Quartile 3					1.10	0.85, 1.41	1.04	0.80, 1.33
Quartile 4					1.11	0.86, 1.42	1.05	0.82, 1.34
*Adult Factors*								
Adult occupational class								
III-NM (Skilled nonmanual)							1.04	0.87, 1.24
III-M (Skilled manual)							1.16	0.94, 1.42
IV (Partly skilled)							1.64	1.34, 2.01
V (Unskilled)							0.66	0.27, 1.42
Age on leaving school, years								
<16							1.16	0.96, 1.39
16							1.08	0.89, 1.31
*General Contextual Effects*								
Sibling level								
Variance	0.245		0.161		0.148		0.182	
MOR	1.60	1.11, 2.11	1.46	1.05, 2.05	1.44	1.05, 2.05	1.50	1.09, 2.04
VPC, %	6.8		4.6		4.3		5.2	
Neighborhood level								
Variance	0.026		0.027		0.030		0.029	
MOR	1.17	1.02, 1.30	1.17	1.02, 1.31	1.18	1.04, 1.31	1.18	1.03, 1.31
VPC, %	0.7		0.8		0.9		0.8	
School level								
Variance	0.020		0.011		0.013		0.012	
MOR	1.14	1.02, 1.28	1.11	1.01, 1.24	1.12	1.01, 1.26	1.11	1.01, 1.25
VPC, %	0.6		0.3		0.4		0.3	
DIC	6,232.45		6,236.43		6,245.23		6,207.61	

Abbreviations: CI, credible interval; DIC, deviance information criterion; MOR, median odds ratio; VPC, variance partition coefficient.

^a^ Mental health was assessed using the 4 items from the General Health Questionnaire, which asks whether, in the past few weeks, respondents have been 1) feeling reasonably happy, all things considered; 2) able to enjoy your normal day-to-day activities; 3) losing confidence in yourself; and 4) feeling unhappy and depressed.

^b^ More than 1 person per room in the home.

^c^ Proportion of homes in the enumeration district without the following 4 amenities: hot water, cold water, fixed bath, and toilet.

^d^ Low proportion of people who owned their own homes in the enumeration district.

^e^ Proportion of children with fathers from professional and managerial occupational classes.

## DISCUSSION

We found a relationship between individual childhood measures of SES and adult self-rated health; the odds of having poor health in adulthood were greater with lower social class in childhood. This relationship was only slightly attenuated by school and neighborhood effects and with the addition of adult occupational class. There was an association of school and neighborhood seen from both the school- and neighborhood-level specific contextual effects of occupational class and the general contextual effects at these levels. These associations are specific to the Aberdeen Children of the 1950s Study cohort, but the methods can be generalized to other contexts.

The median odds ratios showed that school was more strongly associated with adult health than was neighborhood. This study adds to the existing knowledge that the groups of individuals within schools and neighborhoods are associated with adult health over and above individual characteristics (27). Although we found very small general contextual effects at the area level and small general contextual effects at the school level, the specific contextual effects at school and area levels remained associated with poor adult health. Our findings are consistent with those of other studies that have shown contemporaneous school effects on health (3, 4, 10), but our study relates to long-term school influences on self-rated adult health. The context of family in childhood also had an important and lasting association with poor self-rated health and mental health, as shown by the share of the variance at the sibling level (VPC = 4.3%).

A strength of this study is that data from all 12,150 pupils, the entire population of schoolchildren in Aberdeen at that time, were used when calculating the school-level occupational class. This accurate picture of children who attended a particular school is not subject to nonresponse or selection bias. Recall bias is minimized because we used prospective measures of childhood variables.

We used a range of indicators of social position and examined the influence of social position in early life at the individual, family, school, and neighborhood levels to capture the complex environments in which children live. Cross-classified models can account for this hierarchy of the data and model the complex and multifaceted childhood environment (1, 2). It is difficult to measure social position accurately. Although we have adjusted for adult occupational class and age at leaving school, we have not adjusted for adult income nor time spent in each social position as an adult. Because of this imperfect measurement, social patterning may remain. The small residual variance at the school level could be due to the residual clustering of such unmeasured factors within schools.

We have been able to partition the variance into that attributable to siblings and that attributable to individuals. Socioeconomic variables were measured at the family level; children from the same family have the same values (e.g., for paternal occupational class). The inclusion of family as a level means that we have a measure of the unexplained variance at the family level. We found that most of the variation was at the individual level, but family factors were also associated with self-rated adult health. One previous study that was able to do this for contemporary health behaviors found that most of the variance was at the individual and family levels (2).

Limitations of the study include nonresponse to the adult questionnaire; however, unlike previous studies that sampled in adulthood and in which the childhood characteristics of nonresponders were therefore unknown, we had this information. It has been shown that adults whose fathers' social class was higher, as well as adults with higher intelligence quotients, were more likely to respond (14). Therefore, adults with lower paternal social class and lower intelligence quotients are underrepresented in the study. If the relationship between these childhood factors and adult health was different among the nonrespondents, this could affect our findings (16). We were unable to account for adult place of residence. In this Aberdeen cohort, adult place of residence may be very similar to childhood place of residence because most participants remained in the Aberdeen and Grampian areas (15). We found that the neighborhood association with adult health was quite small compared with school and family associations. Other work that used external measures from the census has shown that the proportion of residents of low social class in an area is a good measure of area deprivation across the life course (30). We used information from the 1961 census, and the area measures showed good variability between areas and families.

We used self-reported outcome measures. Self-rated health has been shown to be strongly related to risk of death, with 1 study reporting self-rated health having high power to predict death, equivalent to an objective health measure (31). Mental health is of increasing interest at the population level, and several studies have used self-reported outcomes of mental health (32, 33).

This analysis is intended to identify associations of different aspects of the childhood environment with adult health while acknowledging that association does not necessarily mean causation. Odds ratios for the specific contextual effects are conclusively associated with both outcomes, but their magnitudes are such that they have low discriminatory accuracy (34). In order to develop appropriate public health interventions, measures of discriminatory accuracy, such as receiver operating characteristic curves, should also be considered. These will highlight false positives and false negatives and support better policy recommendations (35, 36).

Findings on the relationship of both social position in childhood and social mobility with adult mental health as measured by the GHQ-4 have been mixed (37–39). Social position at birth and poor mental health in women at age 50 years, but not in men (38), as well as low childhood socioeconomic position have been associated with higher lifetime depression risk (39). When examining life-course socioeconomic position and depression, there was no association between depression and childhood socioeconomic position, but low adult socioeconomic position was associated with higher odds of depression (37). In this study, we found no association with childhood socioeconomic position and little association with adult socioeconomic position for self-reported mental health at age 50 years. Our study was larger than the previous studies (37–39) and used multilevel models, so it was able to distinguish the context of the environment in childhood. We found interesting unexplained variation at the sibling level (median odds ratio = 1.50). This may be due to some unmeasured socioeconomic variables at the family level. It may be due to some aspect of family life—family structure or parenting style—that exhibits a lasting effect on both mental health and self-rated health. It could be that family factors influence the self-rating aspect rather than any clinical aspect of health. The manner in which people identify themselves as having poor health may be influenced by the type of family environment experienced as a child.

There is a need to assess multiple domains of socioeconomic context (39) to allow researchers to study and identify the timing and settings of interventions to address the inequalities that exist across the life course. The World Health Organization's Commission on Social Determinants of Health identified early childhood development as 1 of their action areas and “Healthy Places Healthy People” as another (40). We found that most of the variation in adult health occurs between children and families within areas and schools. Despite this, there is still variation between areas and schools, and school- and area-level factors are of interest. Cross-classified multilevel models have allowed the fitting of models to account for the many environments in which children live and to measure associations between these different environments and adult health.

Both socioeconomic context and composition in early life are important indicators of adult health, even after adjustment for current social position. Policymakers and researchers could focus on early life and childhood family environments to intervene to improve health in later life.

## APPENDIX 1

For a nested hierarchical structure data set, a multilevel logistic regression would be used to model the probability *p_ijkl_* of individual *i* in sibling group *j*, neighborhood *k*, and school *l* having poor self-rated health. This is expressed as

$$\text{logit}(\;p_{ijkl})=\beta_{0}+\beta X_{ijkl}+u_{\;jkl}+v_{kl}+f_{l},$$

where ***X_ijkl_*** is the vector of explanatory variables; *u_jkl_* is normally distributed with variance $\sigma_{u}^{2}$; *v_kl_* is normally distributed with variance $\sigma_{v}^{2}$; and *f_l_* is normally distributed with variance $\sigma_{f}^{2}$.

For the cross-classified design described in this paper, using the notation of Browne (25) to avoid multiple subscripts and estimating the sibling variance only for sibling groups of more than 1, this becomes

$$\text{logit}(\;p_{i})=\beta_{0}+\beta X_{i}+u_{\mathrm{school}(i)}^{\left(4\right)}+u_{\mathrm{neighbourhood}(i)}^{\left(3\right)}+\delta_{j}u_{\mathrm{sibling}\;\mathrm{group}(i)}^{\left(2\right)},$$

where ***X_i_*** is the vector of explanatory variables

$$\;u_{\mathrm{school}(i)}^{\left(4\right)}∼N(0,\sigma_{u(4)}^{2}),$$

$$u_{\mathrm{neighbourhood}(i)}^{\left(3\right)}∼N(0,\sigma_{u(3)}^{2}),$$

and

$$\;u_{\mathrm{sibling}\;\;\mathrm{group}(i)}^{\left(2\right)}∼N(0,\sigma_{u(2)}^{2}),$$

and δ_j_ is an indicator variable for jth sibling group with δ*_j_* = 1 if the sibling group contains more than 1 child, and δ*_j_* = 0 if the sibling group contains only 1 child.

The variance at the sibling level is reparameterized to reflect the fact that most sibling groups contain only 1 individual, and estimating a “variance” for them at this level may not be appropriate. The model used multiplies the residual at the sibling level by 0 (suppressing estimation) if the sibling group contains 1 person and by 1 if the sibling group contains more than 1 person. This has a theoretical basis and is a modification of the technique used by Rasbash et al. (2).

## APPENDIX 2

Variance partition coefficients (VPCs) provide information on the share of the variance at each level. The VPC at each level was calculated using the latent variable method, which assumes a threshold model, and approximating the level-1 (individual) variance by π^2^/3 (≈3.29) (27, 29).

$${\text{VPC}}_{\mathrm{school}}=\frac{\sigma_{u(4)}^{2}}{\left(\sigma_{u(4)}^{2}+\sigma_{u(3)}^{2}+\sigma_{u(2)}^{2}+\pi^{2}/3\right)},$$

$${\text{VPC}}_{\mathrm{neighbourhood}}=\frac{\left(\sigma_{u(3)}^{2}\right)}{\left(\sigma_{u(4)}^{2}+\sigma_{u(3)}^{2}+\sigma_{u(2)}^{2}+\pi^{2}/3\right)},$$

and

$${\text{VPC}}_{\mathrm{sibling}\;\mathrm{group}}=\frac{\left(\sigma_{u(2)}^{2}\right)}{\left(\sigma_{u(4)}^{2}+\sigma_{u(3)}^{2}+\sigma_{u(2)}^{2}+\pi^{2}/3\right)},$$

where $\sigma_{u(4)}^{2}$ is the variance at the school level, $\sigma_{u(3)}^{2}$ is the variance at the neighborhood level, and $\sigma_{u(2)}^{2}$ is the variance at the sibling group level.

The interpretation of the VPCs as calculated above is as follows:

VPC_school_ is the percentage of variance due to differences between schools or the share of the variance attributable to the school level.

VPC_neighborhood_ is the percentage of variance due to differences between areas or the share of the variance attributable to the neighborhood level.

VPC_sibling group_ is the percentage of variance due to differences between sibling groups or the share of the variance attributable to the sibling group level.
